# Practice Makes Efficient: Cortical Alpha Oscillations Are Associated With Improved Golf Putting Performance

**DOI:** 10.1037/spy0000077

**Published:** 2016-11-28

**Authors:** Germano Gallicchio, Andrew Cooke, Christopher Ring

**Affiliations:** 1 School of Sport, Exercise, and Rehabilitation Sciences, University of Birmingham; 2 School of Sport, Health, and Exercise Sciences, Bangor University; 3 School of Sport, Exercise, and Rehabilitation Sciences, University of Birmingham

**Keywords:** alpha oscillations, EEG, golf putting, practice, psychomotor efficiency

## Abstract

Practice of a motor skill results in improved performance and decreased movement awareness. The *psychomotor efficiency* hypothesis proposes that the development of motor expertise through practice is accompanied by physiological refinements whereby irrelevant processes are suppressed and relevant processes are enhanced. The present study employed a test–retest design to evaluate the presence of greater neurophysiological efficiency with practice and mediation analyses to identify the factors accounting for performance improvements, in a golf putting task. Putting performance, movement-specific conscious processing, electroencephalographic alpha power and alpha connectivity were measured from 12 right-handed recreational golfers (age: *M* = 21 years; handicap: *M* = 23) before and after 3 practice sessions. As expected, performance improved and conscious processing decreased with training. Mediation analyses revealed that improvements in performance were partly attributable to increased regional gating of alpha power and reduced cross-regional alpha connectivity. However, changes in conscious processing were not associated with performance improvements. Increased efficiency was manifested at the neurophysiological level as selective inhibition and functional isolation of task-irrelevant cortical regions (temporal regions) and concomitant functional activation of task-relevant regions (central regions). These findings provide preliminary evidence for the development of greater psychomotor efficiency with practice in a precision aiming task.

Practice of a motor skill typically results in improved movement execution and performance. According to the psychomotor efficiency hypothesis (Hatfield & Hillman, 2001), such improvements are accompanied by the suppression of task-irrelevant processes (e.g., diverting resources away from cortical regions that have limited relevance for the task) and the enhancement of task-relevant processes (e.g., redirecting resources to the most important cortical regions for task-performance). At the neurophysiological level, a compelling body of research has found indirect support for this hypothesis by revealing that, while performing precision skills such as golf putting, shooting, and archery, expert athletes manifest greater neural efficiency than novices (for a review, see Cooke, 2013; Hatfield, Haufler, Hung, & Spalding, 2004). By adopting a test-retest design, the aim of the current study was to more directly test the psychomotor efficiency hypothesis. Specifically, we examined (a) whether practice of a motor skill over time leads to neurophysiological adaptations compatible with increased psychomotor efficiency and (b) whether such adaptations account for improvements in movement performance.

Most research relating to neural efficiency in precision sports has examined electroencephalographic (EEG) activity in preparation for action and during movement execution. The EEG measures time-varying changes in voltages from an array of scalp electrodes and reflects postsynaptic potentials in the pyramidal neurons of the cerebral cortex (Nunez & Srinivasan, 2006). The interplay of these potentials generates oscillations at different frequencies, including alpha oscillations (around 8–12 Hz), which are thought to play a major role in shaping the functional architecture of the cortex due to their proposed inhibitory function (Klimesch, 2012). Specifically, the magnitude of alpha oscillations—that is, alpha power—can influence regional activation in the cortex through a gating mechanism whereby resources are diverted away from regions showing higher alpha power (i.e., more inhibition) and toward regions showing lower alpha power (i.e., lower inhibition; Jensen & Mazaheri, 2010).

The study of alpha oscillations in precision sports has revealed that experts display higher alpha power over the temporal regions (e.g., Haufler, Spalding, Santa Maria, & Hatfield, 2000; Janelle et al., 2000) and lower alpha power over the central regions (e.g., Cooke et al., 2014) of the cortex compared to novices while preparing for movement execution. Additionally, experts and novices show different time dynamics of alpha power. For example, Cooke et al. (2014) observed a biphasic pattern of alpha oscillations that was stronger for experts than novices: alpha power showed an initial increase followed by a sudden drop in the last second preceding movement initiation. Taken together these findings suggest the presence of a pattern of cortical activity across the scalp where the timely inhibition of some cortical regions (e.g., temporal) and the lack of inhibition of other regions (e.g., central) can be related to the development of motor expertise.

Complementing the study of the regional and temporal dynamics of alpha power, a few studies have examined the functional connectivity among alpha oscillations across different regions of the cortex. Alpha connectivity between two regions represents the extent to which the alpha activity of those regions is functionally connected (i.e., frequency-specific cortico–cortical communication between different regions). Based on the assumption that alpha reflects inhibition (Klimesch, 2012), alpha connectivity indicates the strength of the functional connection between the inhibition of one region and the inhibition of another region. For example, greater alpha connectivity could be interpreted to reflect two regions engaging in similar and consistent inhibition, whereas lower connectivity may indicate distinct inhibition profiles.

Research in precision sports has revealed that, compared to novices, experts display lower left temporal:frontal alpha connectivity, reflecting a functional disconnection between alpha oscillations of the left temporal region and alpha oscillations of the frontal region (e.g., Gallicchio, Cooke, & Ring, 2016). Building upon the notion that the left temporal and the frontal regions are involved in language and movement planning respectively, reduced left temporal:frontal alpha connectivity has been interpreted as a marker of the selective inhibition of the left-hemisphere and decreased cognitive/verbal interference during preparation for movement execution (Deeny, Hillman, Janelle, & Hatfield, 2003).

More recently, a series of studies has associated left temporal:frontal alpha connectivity with the propensity to consciously monitor and control one’s movements—that is, movement-specific conscious processing—during golf putting (Gallicchio, Cooke, et al., 2016; Zhu, Poolton, Wilson, Maxwell, & Masters, 2011). Three lines of evidence support these views. First, lower left temporal:frontal alpha connectivity in preparation for putting as well as lower putting-related conscious processing were found for expert golfers compared to novices (Gallicchio, Cooke, et al., 2016). Second, individuals who were dispositionally less prone to engage in conscious processing displayed lower left temporal:frontal alpha connectivity prior to putting compared to individuals more prone to engage in conscious processing (Zhu et al., 2011). Third, novice golfers who were trained implicitly, which was associated with lower conscious processing, showed decreased left temporal:frontal alpha connectivity when putting compared to novice golfers who were trained explicitly (Zhu et al., 2011).

Taken together, these findings suggest that decreased left temporal:frontal alpha connectivity and decreased movement-specific conscious processing are features of expertise. This is in line with classic theories of motor skill learning that argue that the development of motor expertise is accompanied by a gradual withdrawal of cognitive analysis and decreased awareness of one’s movements (e.g., Fitts & Posner, 1967). These theories suggest that, following extensive practice, individuals can progress from a cognitive stage, characterized by deliberate and conscious analysis of movement, to an autonomous stage, characterized by automatic control of movement.

While the extant literature argues for greater neural efficiency as expertise develops, some potential limitations still need to be overcome. First, the putative link between expertise and neural efficiency is mostly based on expert–novice differences seen in cross-sectional designs. These findings do not provide a direct test of the hypothesis that practice leads to greater neural efficiency because of the unfeasibility of randomly allocating participants to either the expert or the novice group. For example, it could be that, irrespectively of practice, individuals who show greater neural efficiency are more likely to become experts compared to individuals who show lower neural efficiency. To date, only two studies have examined the effects of practice on neural efficiency using a longitudinal design (Kerick, Douglass, & Hatfield, 2004; Landers et al., 1994). These studies found that performance improvements in archery (Landers et al., 1994) and pistol shooting (Kerick et al., 2004) after three months of training were associated with increased alpha power over the left temporal region of the cortex. However, they did not examine any practice-induced changes in cortical connectivity.

Second, no study to date has examined the neurophysiological factors accounting for the development of expertise. Within-subject mediation analyses (Judd, Kenny, & McClelland, 2001) can be used to examine changes in neural efficiency as a function of performance improvements and thereby shed some light on the mechanisms responsible for the improvements associated with practice.

Third, most studies have employed global measures of performance (e.g., hits vs. misses, distance from the target) that can potentially obscure the individual contribution of distinct parameters involved in movement planning and execution. For example, the movement of a golf ball putted on a flat surface can be conceptualized as a vector having a certain direction and force. Indeed, there is good evidence that there are different neuronal populations that respond selectively to changes in movement direction and force (e.g., Riehle & Requin, 1995). Accordingly, the examination of angle and length errors, respectively associated with movement direction and force, can provide more refined measures of performance that may be differentially sensitive to changes in neural efficiency.

The present exploratory study was designed to address these limitations. Our aims were threefold. First, to describe the neurophysiological adaptations that accompany the development of expertise through practice. Second, to identify neurophysiological mediators that account for changes in performance and movement-specific conscious processing with practice. Third, to evaluate the differential impact of movement direction and force planning on neurophysiological activity. Data were collected in the context of a study designed to examine the efficacy of a neurofeedback training protocol on golf putting performance (Ring, Cooke, Kavussanu, McIntyre, & Masters, 2015). Here we report new analyses that were conducted on the data of the control group who underwent putting training sessions while receiving sham neurofeedback (i.e., who did not receive genuine feedback of cortical activity). We expected that performance would improve with practice and that these improvements would be mediated by increased regional gating of alpha power, reduced cross-regional alpha connectivity, as well as reduced movement-specific conscious processing, in accord with the predictions of the psychomotor efficiency hypothesis.

## Method

### Participants

Twelve right-handed male recreational golfers took part in this study (age: *M* = 21.00, *SD* = 2.52 years). The participants reported a mean golf experience of 4.63 years (*SD* = 2.89) and a mean golf handicap of 23.33 (*SD* = 4.62). All participants provided informed consent.

### Putting Task

Golf balls (diameter 4.7 cm) were putted on an artificial flat putting surface (Turftiles) to a hole (diameter 10.8 cm) at a distance of 2.4 m, using a blade-style putter (length 90 cm). The participants were instructed to get each ball “ideally in the hole, but if unsuccessful, to make them finish as close to the hole as possible.”

### Training

In each 1-hr training session participants practiced putting. Participants wore a cap with one frontal scalp electrode and reference and ground electrodes placed on the left and right mastoids respectively. They were instructed to try to regulate the pitch of a tone by changing their brain activity while preparing for putting and then to putt the ball when the tone was silenced. Specifically, they would stand over the ball and hear the pitch of a tone increase and decrease, and occasionally go silent for 1.5 s, which was a cue to putt. In reality, the tone was independent of their brain activity (i.e., sham neurofeedback), and was yoked to an experimental participant who received genuine neurofeedback: thus the sham feedback participants acted as controls in Ring et al. (2015). Each training session comprised 12 blocks of 5 min each.

### Procedure

A test–retest design was employed, with participants visiting the laboratory on 5 different days: putting task on Day 1 (i.e., test), training on Days 2–4, putting task on Day 5 (i.e., retest). On average, the test–retest interval was 8.17 (*SD* = 5.24) days and the final training session to retest session interval was 2.00 (*SD* = 2.59) days. In the test and retest sessions, participants were instrumented for EEG recording, instructed, then completed 20 familiarization putts followed by 50 test putts. In each of the three training sessions separating the test and retest sessions, participants completed a mean of 181.25 (*SD* = 52.25) practice putts. Thus, the total number of putts in training was 543.75 (*SD* = 127.01). The study protocol was approved by the local research ethics committee.

### EEG Recording

In the test and retest sessions, 32 active electrodes were positioned on the scalp, according to the 10–20 system, at Fp1, Fp2, AF3, AF4, F7, F3, Fz, F4, F8, FC5, FC1, FC2, FC6, T7, C3, Cz, C4, T8, CP5, CP1, CP2, CP6, P7, P3, Pz, P4, P8, PO3, PO4, O1, Oz, and O2. In addition, four active electrodes were placed at the bottom and at the outer canthus of both eyes. Common mode sense and driven right leg electrodes were used to enhance the common mode rejection ratio of the signal. The signal was amplified and digitized at 512 Hz with 24-bit resolution, using the ActiveTwo recording system (Biosemi, the Netherlands). Signals were down-sampled offline to 256 Hz, 1- to 35-Hz band-pass filtered (Finite Impulse Response, Order 512), and rereferenced to the average of all EEG channels. Channels with bad signals were removed and interpolated prior to averaging. Nonneural activity was minimized using the Artifact Subspace Reconstruction plugin for EEGLAB (Delorme & Makeig, 2004). Epochs were extracted from −3.25 to +1.25 s relative to the initiation of the backswing, which was triggered when the putter head broke the beam of an optical sensor interfaced with the ActiveTwo recording system.

Time–frequency decomposition was performed through short-time fast Fourier transform (FFT) on 33 overlapping segments each of the duration of 0.5 s and linearly spaced with center points ranging from −3 to +1 s. Prior to FFT, each segment was also Hanning-windowed to taper both ends to 0 and then 0-padded to reach 2-s duration. This procedure generated complex-valued FFT coefficients in the time–frequency plane with a precision of 0.125 s and 0.5 Hz. Six regions of interest (ROIs) were identified: left temporal (FC5, T7, CP5), left central (FC1, C3, CP1), frontal (F3, Fz, F4), right central (FC2, C4, CP2), right temporal (FC6, T8, CP6), and occipital (O1, Oz, O2). Signal processing was performed using the EEGLAB toolbox (Delorme & Makeig, 2004) and MATLAB.

### Measures

#### Putting performance

The number of holed putts out of 50 was recorded in the test and retest sessions. Additionally, three performance errors—radial (cm), angle (degrees), and length (cm) errors (see Figure S1 in the supplemental materials)—were computed for each putt using a camera system (Neumann & Thomas, 2008) and averaged (geometric mean) to yield measures for the test and retest sessions.

#### Alpha power

Power (μV^2^) was computed in the time–frequency plane separately for each channel and trial (i.e., putt) as the product between each FFT coefficient and its complex conjugate (i.e., equivalent to amplitude squared). Importantly, no baseline was employed. Instead, skewness and interindividual differences in the power density distributions were dealt with by employing a median-scaled transformation (cf. Gallicchio, Finkenzeller, Sattlecker, Lindinger, & Hoedlmoser, 2016): each participant’s values were scaled by their median and then log-transformed (10 · log_10_). This procedure meant that power was normally distributed with a mean of zero for each participant, without altering within-subject relations. Power was then averaged across time (−3 to −2 s, −2 to −1 s, −1 to 0 s, 0 to +1 s, where zero represents initiation of the backswing), channels (ROIs), putts, and frequency (10–12 Hz) to yield estimates of alpha oscillatory power in each session (test, retest). Alpha is typically around 8–12 Hz; however, we focused on the upper portion of this range, (i.e., 10–12 Hz) on the basis of spectral features that were evident in the current data (see Figure S4 in the supplemental materials).

#### Alpha connectivity

Intersite phase clustering (ISPC) was computed as the length of the complex-valued resultant of cross-trial clustering of unitary complex vectors having as angle the phase difference between channel pairs for each point of the time–frequency plane (M. X. Cohen, 2014; Lachaux, Rodriguez, Martinerie, & Varela, 1999). ISPC measures the phase lag consistency across trials (i.e., putts) between two channels independently from their power and reflects the functional connectivity between the oscillatory activity of two underlying cortical regions, with values ranging from 0 (*no connectivity*) to 1 (*perfect connectivity*). The impact of volume conduction on connectivity was examined by taking the absolute imaginary part of the ISPC (imISPC; cf. Nolte et al., 2004). Like ISPC, imISPC reflects functional connectivity with values ranging from 0 to 1; however, imISPC is insensitive to instantaneous connectivity (i.e., 0- or π-lagged) and, therefore, values are much smaller than ISPC. No baselines were used. Instead, to normalize their density distributions, ISPC and imISPC were Fisher *Z* transformed (inverse hyperbolic tangent); values could range then from 0 to ∞. Values were then averaged (arithmetic mean) across time (−3 to −2 s, −2 to −1 s, −1 to 0 s, 0 to +1 s), channel (ROI) pairs, and frequency (10–12 Hz) to yield estimates of alpha connectivity in each session (test, retest).

#### Conscious processing

Self-reported conscious processing was measured immediately after completing the putting task in the test and retest sessions using a putting-specific version (Cooke, Kavussanu, McIntyre, Boardley, & Ring, 2011; Vine, Moore, Cooke, Ring, & Wilson, 2013) of the conscious motor processing subscale of the Movement Specific Reinvestment Scale (Orrell, Masters, & Eves, 2009). This scale consists of six items scored on a 5-point Likert scale (1 = *never*, 3 = *sometimes*, 5 = *always*) related to the feeling of awareness of the kinematics involved in execution of the putt and thoughts about putt outcome. The six items were averaged to generate a single scale score. Past research (Cooke et al., 2011; Vine et al., 2013) has established the reliability (α = .81–.88) and validity of the putting-specific version of the conscious motor processing subscale of the Movement Specific Reinvestment Scale.

### Statistical Analyses

#### Performance and conscious processing

Changes from test to retest in putting performance and conscious processing were examined by paired-sample *t* tests. Within each session the relation between the number of holed putts and the three performance errors was examined through Pearson’s correlations.

#### Alpha power and connectivity

Power was subjected to a 2 (Session: test, retest) × 6 (ROI: left temporal, left central, frontal, right central, right temporal, occipital) × 4 (Time: −3 to −2, −2 to −1, −1 to 0, 0 to +1 s) analysis of variance (ANOVA). In addition, contrast analyses were performed to examine changes in power over time. ISPC and imISPC were each subjected to 2 (Session) × 4 (Time) ANOVAs, conducted separately on each of two ROI pairs (left temporal:frontal, right temporal:frontal), chosen on the basis of previous literature (Deeny, Haufler, Saffer, & Hatfield 2009; Deeny et al., 2003; Gallicchio, Cooke, et al., 2016; Zhu et al., 2011). The multivariate solution was reported in the ANOVAs where appropriate (Vasey & Thayer, 1987). Significant main effects were interrogated using post hoc testing. Partial eta-squared (η_p_^2^) and *r*^2^ are reported as measures of effect size: values of .02, .13, and .26 were taken to reflect small, medium, and large effects, respectively (J. Cohen, 1992).

#### Mediation

Mediation analyses were conducted to test whether changes across sessions in the number of holed putts could be accounted for by changes in performance errors, conscious processing, alpha power, and alpha connectivity. We also tested whether changes in conscious processing could be attributed to changes in alpha power and connectivity. We used the procedure described by Judd et al. (2001) for repeated-measures designs: multiple regression was used to predict the test to retest change in the dependent variable based on the test to retest change in the potential mediator variable, while controlling for its mean-centered sum. Full mediation can be inferred when the regression coefficient associated with the change in the mediator variable is significant (i.e., *p* < .05), and partial mediation is inferred when the coefficient associated with the intercept is also significant. The following strategy was adopted to reduce the likelihood of Type I errors: We first assessed whether the change in the number of holed putts was mediated by changes in any of the potential mediator variables, and only if this was the case were mediation analyses conducted on the changes in the performance errors and conscious processing.

## Results

### Putting Performance

Overall, every putting performance measure improved with training from test to retest (Table 1). However, there were considerable individual differences: Not all participants improved equally and in fact a few got worse (see Figure S2 in the supplemental materials). The number of holed putts was highly negatively correlated with the three performance errors (*r*s = –.77 to –.92, *p*s < .003), with angle error the highest (see Table S1 in the supplemental materials).[Table-anchor tbl1]

### Alpha Power

**Figure 1 fig1:**
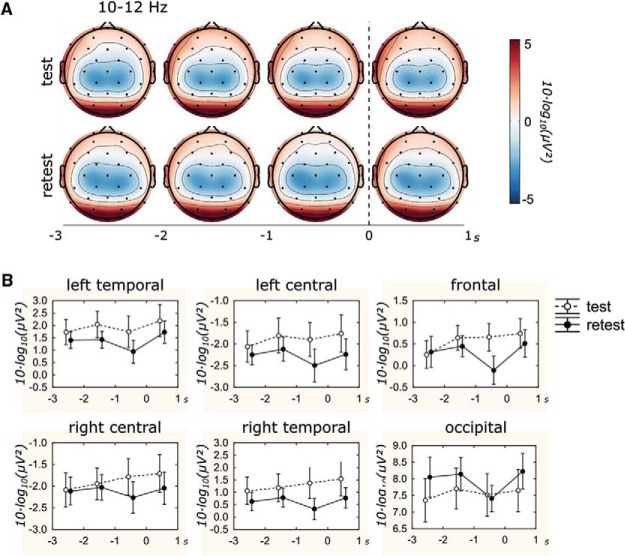
(A) Scalp maps representing alpha power [10 · log10(μV^2^)] averaged across participants, as a function of session (test, retest), time (−3 to +1 s), and channel. (B) Alpha power [10 · log10(μV^2^)] averaged across participants, as a function of session (test, retest) and time (−3 to +1 s) in the six regions. Error bars represent the standard error of the mean. See the online article for the color version of this figure.

The 2 (Session) × 6 (ROI) × 4 (Time) ANOVA conducted on EEG power revealed a large main effect of ROI, *F*(5, 7) = 105.49, *p* < .001, η_p_^2^ = .987. Post hoc Scheffé tests indicated (*p* < .001) that power was higher in the occipital than left/right temporal and frontal regions, which, in turn, were higher than left/right central regions (Figure 1A). Power tended to be lower in the retest session than the test session (Figure 1B), *F*(1, 11) = 0.78, *p* = .40, η_p_^2^ = .066, in all regions (left temporal Δ = −0.55; left central Δ = −0.40; frontal Δ = −0.28; right central Δ = −0.23; right temporal Δ = −0.66) except the occipital region (Δ = 0.40). Although no clear omnibus time effect was evident, *F*(3, 9) = 2.93, *p* = .09, η_p_^2^ = .494, the effect size was large, and, therefore, we performed contrast analyses to characterize the a priori predicted changes in power in the moments surrounding movement; a series of 4 (Time) ANOVAs (contrast codes: 0, 1, −2, 1) were conducted separately for each session and ROI. This quadratic trend was not displayed in the test session, *F*s(1,11) = 0.02–0.74, *p*s = .41–.89, η_p_^2^s = .002–.063,with the sole exception of the left temporal region, *F*(1, 11) = 4.10, *p* = .07, η_p_^2^ = .271, but was clearly evident in all regions in the retest session, *F*s(1, 11) = 12.57–4.01, *p*s = .005–.07, η_p_^2^s = .267–.533. This implies a practice-induced time-varying change in alpha power, characterized mainly by a reduction in power during the final second before movement following practice during the retest session (Figure 1B).[Fig-anchor fig1]

### Alpha Connectivity

**Figure 2 fig2:**
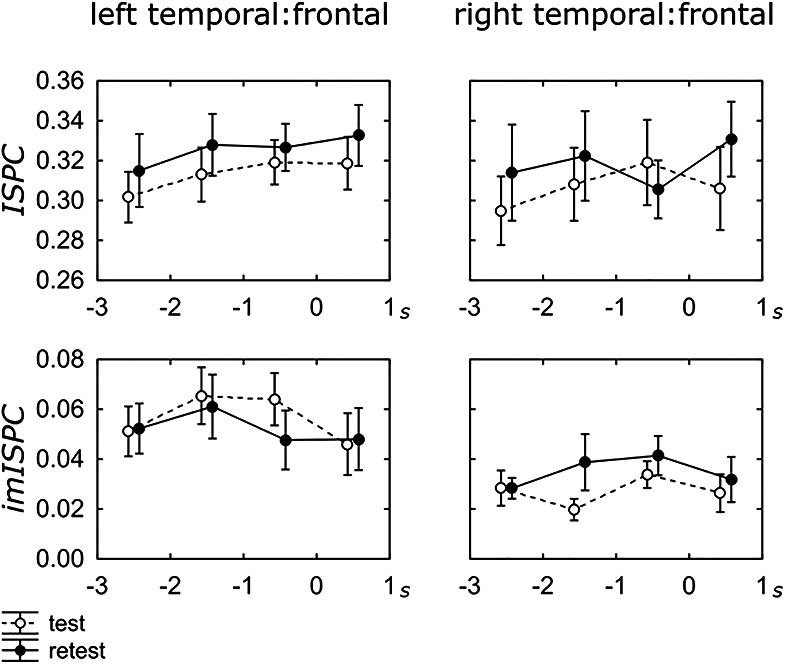
Left/right temporal:frontal alpha ISPC and imISPC averaged across participants as a function of session (test, retest) and time (−3 to +1 s). Error bars represent the standard error of the mean.

The 2 (Session) × 4 (Time) ANOVAs on the left temporal:frontal connectivity indices (Figure 2) revealed no main effects for session, ISPC: Δ = 0.01, *F*(1, 11) = 1.02, *p* = .34, η_p_^2^ = .085; imISPC: Δ = −0.004, *F*(1, 11) = 0.35, *p* = .57, η_p_^2^ = .031, or time, ISPC: *F*(3, 9) = 0.77, *p* = .54, η_p_^2^ = .203; imISPC, *F*(3, 9) = 3.46, *p* = .06, η_p_^2^ = .536. Similarly, no effects emerged with right temporal:frontal connectivity (Figure 2) as a function of session, ISPC: Δ = 0.01, *F*(1, 11) = 0.75, *p* = .41, η_p_^2^ = .064; imISPC: Δ = 0.008, *F*(1, 11) = 2.512, *p* = .14, η_p_^2^ = .186, and time, ISPC: *F*(3, 9) = 0.63, *p* = .61, η_p_^2^ = .174; imISPC: *F*(3, 9) = 0.69, *p* = .58, η_p_^2^ = .187. No session by time interactions emerged. Finally, the results from all ROI pairs are reported in the supplemental materials (Figure S5) for interested readers.[Fig-anchor fig2]

### Conscious Processing

Overall conscious processing decreased from test (*M* = 3.88, *SD* = 0.20) to retest (*M* = 3.36, *SD* = 0.24), *t*(11) = 2.59, *p* = .03, *r*^2^ = .378. Again, there were large individual differences in the extent of this change, with four participants opposing the trend by reporting the same or greater conscious processing after training (supplemental materials, Figure S3).

### Mediators of the Change in Putting Performance

Putting performance improved with practice. On average, participants holed 4.08 more balls (i.e., an 8.2% improvement) in the retest session compared to the test session. Judd et al.’s (2001) regression-based within-subject mediation analyses indicated that this improvement was fully mediated by the reduction in angle error from test to retest (*b* = −9.82, *p* = .008); the intercept (*a* = 1.89, *p* = .21) indicated that, had angle error not changed from test to retest, the improvement would have been reduced to only 1.89 additional holed putts, which represents a nonsignificant change in performance. Neither radial error (*b* = −0.88, *p* = .06) nor length error (*b* = −0.81, *p* = .17) mediated performance improvement. Further, conscious processing did not mediate the change in performance (*b* = −1.23, *p* = .70).

**Figure 3 fig3:**
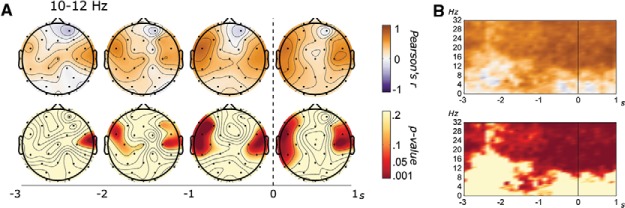
(A) Scalp maps representing Pearson’s correlations conducted on the intersession change scores between the number of holed putts and alpha power, as a function of time (−3 to +1 s) and channel. (B) Time–frequency plots representing Pearson’s correlations conducted on the intersession change scores between the left temporal alpha power [10 · log10(μV^2^)] and the number of holed putts, as a function of time (−3 to +1 s) and frequency (0 to 32 Hz). See the online article for the color version of this figure.

In terms of alpha power, the improvement in putting performance was partially mediated by the change in left temporal power in the seconds surrounding backswing initiation (−1 to 0 s: *b* = 2.46, *p* = .04; 0 to 1 s: *b* = 2.07, *p* = .04; see Figure 3). Because power tended to decrease with practice (Figure 1B), smaller reductions in left temporal power from test to retest were associated with larger improvements in performance. Based on the associated intercepts (−1 to 0 s: *a* = 6.07, *p* = .005; 0 to 1 s: *a* = 5.04, *p* = .01), this means that an individual who increased their left temporal power from test to retest in the second before backswing initiation would be predicted to hole at least two more putts whereas someone who increased power from test to retest in the second after initiation would be predicted to hole at least one more putt. Furthermore, left temporal power within the −1 to 0 s interval also partially mediated the reduction in angle (*b* = −0.19, *p* = .03) but not radial (*b* = −1.72, *p* = .06) or length (*b* = −1.33, *p* = .09) errors (supplemental materials, Figure S6).[Fig-anchor fig3]

In terms of alpha connectivity, putting performance was partially mediated by the intersession change in left temporal:frontal ISPC within the −2 to −1 s interval (*b* = −120.60, *p* = .01). Because ISPC tended to increase with practice (Figure 2), smaller increases in left temporal:frontal connectivity from test to retest were associated with larger improvements in putting performance. Based on the intercept (*a* = 5.88, *p* = .004), performance would be predicted to improve by at least two more holed putts if left temporal:frontal ISPC decreased within this time interval. The same analysis conducted on imISPC also revealed a negative relation, (*b* = −53.02, *p* = .28). Furthermore, left temporal:frontal ISPC within the −2 to −1 s interval also partially mediated the reduction in angle (*b* = 6.35, *p* = .05), but not radial (*b* = 56.97, *p* = .13) and length (*b* = 35.52, *p* = .28) errors.

Right temporal:frontal ISPC and imISPC did not mediate the improvement in putting performance (*p*s = .19–.93). Lastly, mediation analyses on all ROI pairs (supplemental materials, Figure S7A–B) indicated that the relation between smaller increases in left temporal:frontal ISPC and greater performance improvement extended to a network linking the left temporal region to the other cortical regions.

### Mediators of the Change in Conscious Processing

On average, participants reported less conscious processing (Δ = −0.52) from test to retest. This reduction in conscious processing was fully mediated (*a* = −0.34, *p* = .09) by the change in left temporal:frontal ISPC within the −2 to −1 s interval (*b* = −11.87, *p* = .03), whereby decreases in conscious processing were associated with increases in ISPC. Finally, the mediation analyses involving all ROI pairs (supplemental materials, Figure S7C, D) showed that changes in conscious processing were related to changes in connectivity across a broad network of cortical regions.

## Discussion

Performance improved from test to retest. That retention was assessed a couple of days after the end of training provided evidence for motor learning (e.g., Salmoni, Schmidt, & Walter, 1984). The primary aim of this exploratory study was to identify the neurophysiological factors that mediate changes in motor performance with practice. Improvements in golf putting performance from before (test) to after (retest) completing three training sessions were mediated by EEG alpha power and alpha connectivity in preparation for putting but not by self-reported conscious processing.

### Alpha Power

Spectral analyses revealed a distinct 10- to 12-Hz peak compatible with the alpha rhythm (see supplemental materials, Figure S4), and therefore activity within this frequency range was interpreted as reflecting cortical alpha oscillations. Alpha activity was displayed across the different regions of the cortex in a focal pattern: power was lowest over the central regions, medium over the temporal regions, and highest over the occipital region. In line with the *gating-by-inhibition hypothesis* (Jensen & Mazaheri, 2010), the observed regional pattern suggests that neuronal resources were taken away from occipital and temporal regions (i.e., highest inhibition) and diverted toward the central regions (i.e., lowest inhibition) during movement preparation. This focal pattern, which was evident in both test and retest sessions, could reflect the prior practice history of our participants, who were all experienced golfers, and therefore had already developed a degree of psychomotor efficiency related to the putting movement.

Efficiency-based changes in alpha power due to training can be inferred from our mediation analyses. Importantly, they suggested that participants who were able to sustain a relatively higher power in the temporal regions from test to retest in the seconds surrounding movement improved their putting performance the most. This effect was localized to the left (and to a lesser extent, the right) temporal region and can be interpreted in terms of alpha gating: increased inhibition in regions not directly involved in putting-relevant processing is beneficial to putting. That this effect was absent in the occipital region is most likely because occipital inhibition was already the strongest among the regions examined and tended to strengthen further with training. In other words, it is likely that there was a ceiling effect for occipital alpha, whereby further increases did not benefit performance.

It is also worth noting that while a relatively higher level of temporal alpha power was beneficial, practice also prompted a decrease in power, especially at the frontal region, in the final second preceding movement. This quadratic trend for time-varying alpha power in the retest session could be interpreted as reflecting the timely allocation of resources to putting-relevant processing (Cooke et al., 2015). Indeed, this quadratic pattern is consistent with previous research and has been associated with expertise and successful performance in experts (Babiloni et al., 2008; Cooke et al., 2014). However, as this quadratic decrease in alpha power at retest did not mediate changes in performance, the inhibition of irrelevant cortical regions seems to have been more important for performance improvement than the timely activation of relevant ones. This remains a topic for future research, which may consider variables such as task and experience as potential moderators of any effects.

### Alpha Connectivity

Functional connectivity was examined between the temporal and frontal regions using two indices based on the consistency of cross-regional phase lag across trials: ISPC and imISPC. The latter is a conservative version of the former that is not biased by volume conduction. The fact that 10–12 Hz imISPC was nonzero (see Figure 2) indicated the likely presence of genuine alpha connectivity. Neither connectivity index changed across the time intervals or from test to retest. However, mediation analyses suggested that greater improvements in performance from test to retest were achieved by participants displaying relatively lower left temporal:frontal connectivity a couple of seconds before putt initiation. Low left temporal:frontal alpha connectivity has been associated with expertise and successful putting performance in experts (Babiloni et al., 2011; Gallicchio, Cooke, et al., 2016). At the neurophysiological level, lower connectivity represents a stronger disconnection between the two signals—that is, left temporal alpha and frontal alpha—provided that the two signals are not projections of the same source generator because of volume conduction within the head.

The additional analyses performed on a wider network of regions (supplemental materials, Figure S7) revealed that performance improvements were not exclusively associated with a stronger disconnection of alpha activity between left temporal and frontal regions. Rather, it is evident that improved performance was associated with a functional isolation of left temporal alpha from many other regional alpha activities. Taken together, these analyses provide preliminary support for our hypothesis that improvements in performance with practice would be mediated by reduced connectivity (i.e., less cortico–cortical communication) between alpha oscillations in the left temporal region and other regions of the cortex, including the frontal region (cf. Deeny et al., 2003; Gallicchio, Cooke, et al., 2016; Zhu et al., 2011).

### Conscious Processing

Movement-specific conscious processing decreased and performance improved with practice, in line with the classic theories of motor skill learning (e.g., Fitts & Posner, 1967). However, mediation analyses did not support the putative link between decreased conscious processing and performance improvement. Similarly, Malhotra, Poolton, Wilson, Omuro, and Masters (2015) also found no relation between improvements in putting performance and changes in conscious processing with training. It should be noted that these two null findings reflect the absence of a linear relation; however, our analyses indicate a curvilinear relationship: participants who reported a moderate decrease in conscious processing improved more than those who reported a large decrease, no change, and even a small increase in conscious processing (supplemental materials, Figure S8). It has been increasingly recognized that conscious processing does not always negatively impact performance but can foster performance improvements in experts (Toner & Moran, 2014) and novices (Malhotra et al., 2015). Given these findings it would be fruitful for future research to seek to identify optimal levels of conscious processing as a function of factors such as task, expertise and personality. Such research should also consider subcomponents of conscious processing, for instance, distinguishing conscious monitoring and conscious control (Toner & Moran, 2011), particularly when they are about to putt, which should be able to paint a better picture of what individuals attend to in the moments before movement initiation.

Mediation analyses suggested that decreases in conscious processing from test to retest were associated with increases in alpha connectivity across a network involving all cortical regions examined (supplemental materials, Figure S7). Higher connectivity represents a stronger connection between the alpha oscillations, and therefore suggests that decreased movement-specific conscious processing or awareness of one’s movements is associated with multiple cortical regions engaging in similar and consistent inhibition (cf. Baars, 2002). This interpretation awaits confirmation.

### Performance Errors

The analyses of the three performance metrics—that is, radial, angle, and length errors—revealed that improvements in the number of holed putts with practice was largely due to reductions in angle error rather than radial or length errors. This finding suggests that a more precise alignment of the putter head with the ball at the moment of impact is more beneficial to putting outcome than appropriate impact velocity (Cooke, Kavussanu, McIntyre, & Ring, 2010). Additionally, all of the significant associations observed between EEG activity and putting performance errors were found for angle error, suggesting that programming of movement direction is better reflected in alpha activity than movement force. Although there is evidence that movement direction and force are selectively coded by different neuronal populations (e.g., Riehle & Requin, 1995), future research is needed to clarify the relationship between alpha oscillations, on the one hand, and programming of movement parameters, on the other hand.

### Limitations and Future Research

The current study yielded some novel and important findings regarding the causal relations among practice, cortical efficiency, conscious processing and performance. However, their interpretation should be considered in light of potential limitations. First, although the putting task was completed under ecologically valid conditions, the training cannot be considered a form of discovery learning because participants received sham neurofeedback. Moreover, we did not employ a control group who did not receive any form of neurofeedback. We cannot determine the impact of the current training protocol and therefore future research should consider replicating our findings using other forms of training, including discovery learning, and appropriate control groups.

Second, we refrained from interpreting activity in different cortical regions in terms of specific cognitive processes because we did not measure nor manipulate cognition directly. We acknowledge that the presence of a certain regional activation makes some cognitive processes more likely to be involved than others, however, we avoided reverse inference (Poldrack, 2006) and postponed interpretation. Indeed, it would be worth studying the relation between regional activation and cognitive processes using experimental designs where cognition is manipulated (rather than simply measured) in the context of precision aiming.

Third, the use of spectral decomposition on (inherently nonstationary) EEG signals implies that power is likely to be greater than 0 at any unfiltered frequency, irrespectively of the presence of actual neural generators oscillating at that frequency. The distinct 10–12 Hz power peak in the group-averaged frequency plots (see supplemental materials, Figure S4) supported the likely presence of cortical oscillations within this frequency band. However, the use of a fixed range did not account for individual variations. Future studies could individually adjust these ranges to obtain greater specificity and sensitivity (cf. Klimesch, 1999).

Fourth, we considered measures of alpha as candidates to mediate the main effect of session on performance despite having nonsignificant main effects themselves. This strategy is in line with current guidelines recommending that mediation only requires the existence of an effect to be mediated (i.e., change in performance) for that effect to be indirectly influenced by the mediator variables (e.g., alpha power; Preacher & Hayes, 2004). Our approach satisfies these criteria, nonetheless, we did not manipulate any of the mediator variables, and therefore the outcome variable (i.e., performance) may have influenced the mediatior variables (Cooke et al., 2015). It would be useful to replicate these analyses in larger samples with more statistical power where the mediators are manipulated independently of the outcome variables, using, for instance, brain stimulation or neurofeedback training.

Fifth, the greater relative importance of the angle error over radial and length errors is potentially biased by the presence of an actual hole, which may have influenced our performance measurements, particularly in regards to length error. For example, balls can be redirected by the hole (e.g., a lip out) and most balls that dropped into the hole would otherwise have rolled past the hole had the hole not been present, introducing variability that cannot be accounted for by the measurements. Future studies could use a mark on the mat instead of a hole to overcome this limitation.

Finally, we only tested experienced golfers that arguably lay somewhere between the cognitive and the autonomous stage of learning (cf. Fitts & Posner, 1967). Given that the particular stage of learning that the individual is in may moderate the adaptations in alpha gating and connectivity with training, future research could examine these learning-related adaptations in novices and experts as well as experienced individuals.

## Conclusions

This exploratory study provides preliminary evidence that practice of a motor skill leads to neurophysiological adaptations compatible with the psychomotor efficiency hypothesis (Hatfield & Hillman, 2001). Efficiency was manifested as selective inhibition and functional isolation of task-irrelevant cortical regions and concomitant functional activation of task-relevant regions. Our findings suggest that processing in broadly central regions (cf. Andersen & Buneo, 2002; Desmurget et al., 2009) is more important than processing in temporal regions (cf. Kerick et al., 2001) while performing a precision aiming task, such as golf putting. These findings imply that larger improvements in precision aiming performance with practice may be achieved by employing training protocols that foster suppression of task-irrelevant processes.

## Supplementary Material

10.1037/spy0000077.supp

## Figures and Tables

**Table 1 tbl1:** Descriptive Statistics of Putting Performance as a Function of Session Together With the Results of the Paired-Sample T Tests

Performance measures	Test *M* (*SD*)	Retest *M* (*SD*)	*t*(11)	*p*	*r*^2^
Holed putts (0–50)	12.17 (2.39)	16.25 (2.97)	2.18	.05	.301
Radial error (cm)	10.95 (1.59)	8.05 (1.23)	2.26	.04	.317
Angle error (degrees)	1.39 (.12)	1.17 (.14)	1.74	.11	.215
Length error (cm)	8.80 (1.27)	6.42 (.95)	2.22	.05	.310
