# Supplementary material for: Practice Makes Efficient: Cortical Alpha Oscillations Are Associated With Improved Golf Putting Performance
Source: Sport Exerc Perform Psychol. 2016 Nov 28;6(1):89–102. doi: 10.1037/spy0000077 (PMC5506342; doi:10.1037/spy0000077)
Supplement: Supplementary file 1 [file FigureS2.pdf]

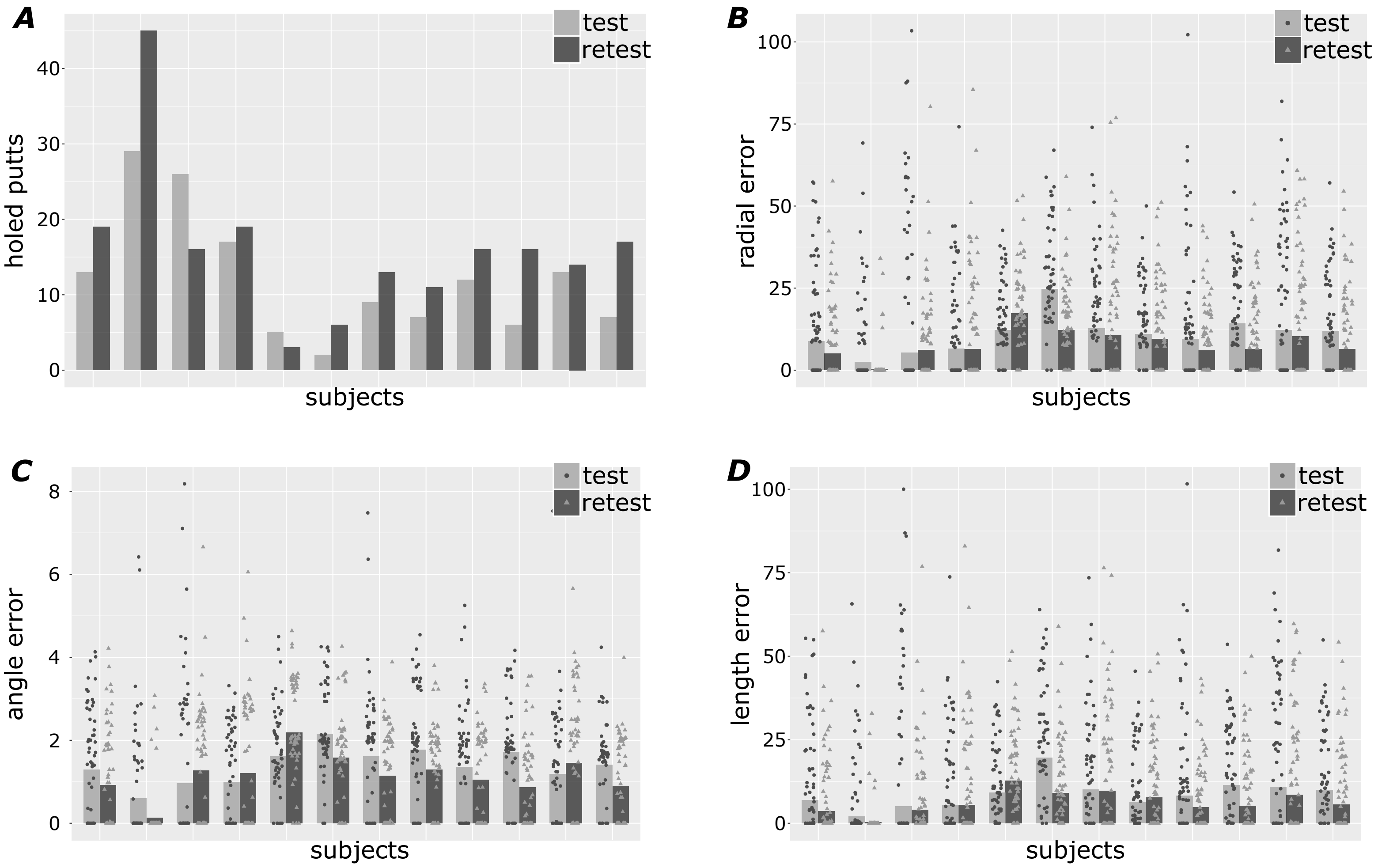

Figure S2.

A: Number of holed putts for each participant as a function of session (test, retest). **B, C, D:** Single-putt values (marks) and their geometric mean (bars) representing radial, angle, and length errors for each participants as a function of session (test, retest).
