# Supplementary figures and images for: Practice Makes Efficient: Cortical Alpha Oscillations Are Associated With Improved Golf Putting Performance
Source: Sport Exerc Perform Psychol. 2016 Nov 28;6(1):89–102. doi: 10.1037/spy0000077 (PMC5506342; doi:10.1037/spy0000077)

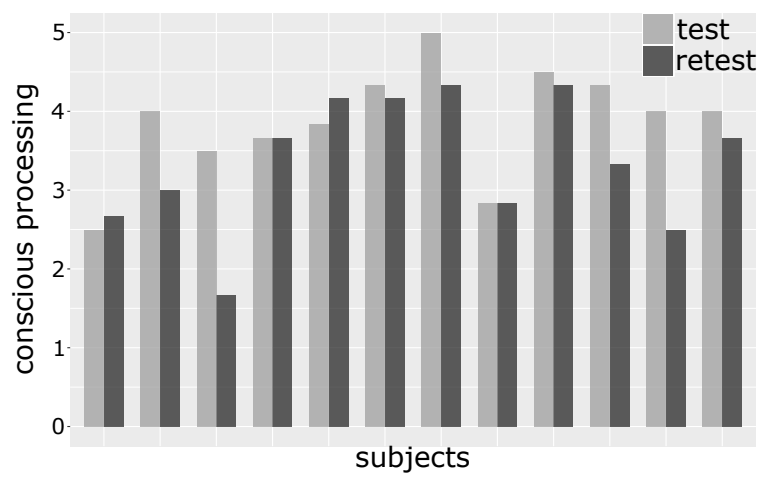

*Figure S3.*  
Conscious processing for each participant as a function of session (test, retest).

Supplement: Supplementary file 2 [file FigureS3.pdf]

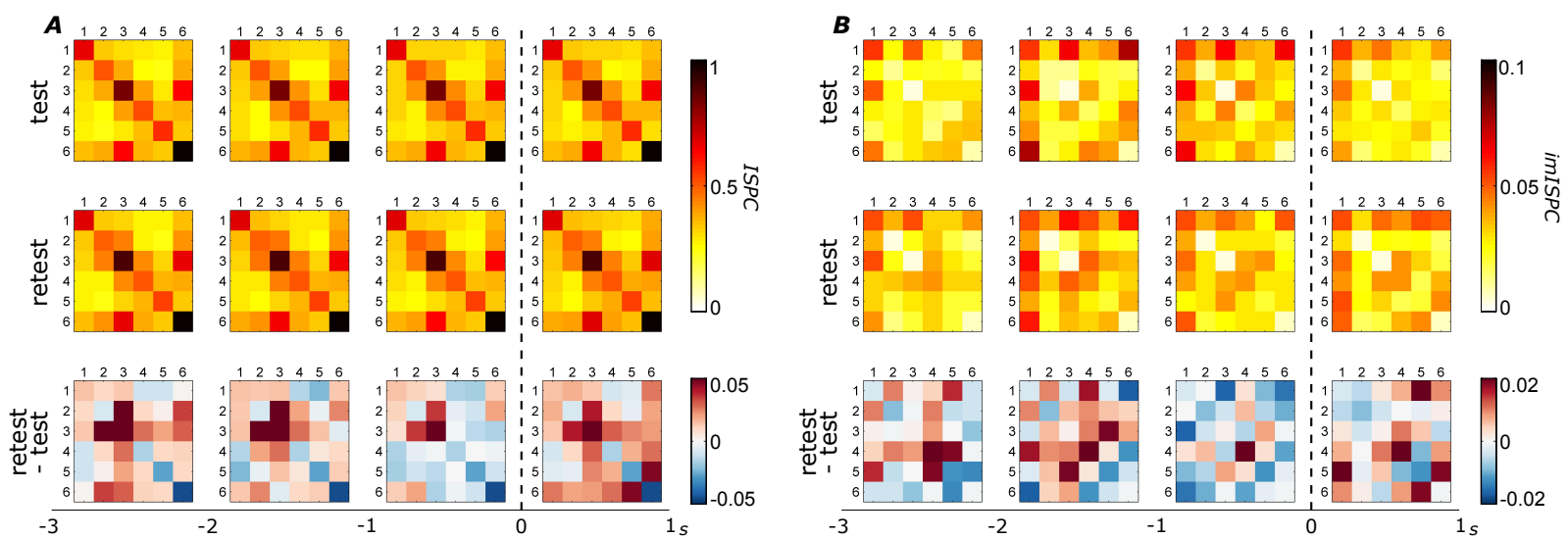

*Figure S5.*

Supplement: Supplementary file 8 [file FigureS5.pdf]
