# Supplementary material for: Practice Makes Efficient: Cortical Alpha Oscillations Are Associated With Improved Golf Putting Performance
Source: Sport Exerc Perform Psychol. 2016 Nov 28;6(1):89–102. doi: 10.1037/spy0000077 (PMC5506342; doi:10.1037/spy0000077)
Supplement: Supplementary file 3 [file spy-0226_SPY-2016-0069_SUPPL.zip › spy004160226so9.docx]

**Supplemental Materials**

**Practice Makes Efficient: Cortical Alpha Oscillations Are Associated With Improved Golf Putting Performance**

**by G. Gallicchio et al., 2016, *Sport, Exercise, and Performance Psychology***

**http://dx.doi.org/10.1037/spy0000077**

*Table S1.* Pearson's correlations (and p-values associated) between the number of holed putts and the performance errors (radial, angle, length) within each session.

|  | Test | | Retest | |
| --- | --- | --- | --- | --- |
|  | ***r*** | ***p*** | ***r*** | ***p*** |
| radial error | -.83 | < .001 | -.87 | < .001 |
| angle error | -.92 | < .001 | -.89 | < .001 |
| length error | -.77 | .003 | -.85 | < .001 |
