# Supplementary material for: Practice Makes Efficient: Cortical Alpha Oscillations Are Associated With Improved Golf Putting Performance
Source: Sport Exerc Perform Psychol. 2016 Nov 28;6(1):89–102. doi: 10.1037/spy0000077 (PMC5506342; doi:10.1037/spy0000077)
Supplement: Supplementary file 4 [file FigureS1.pdf]

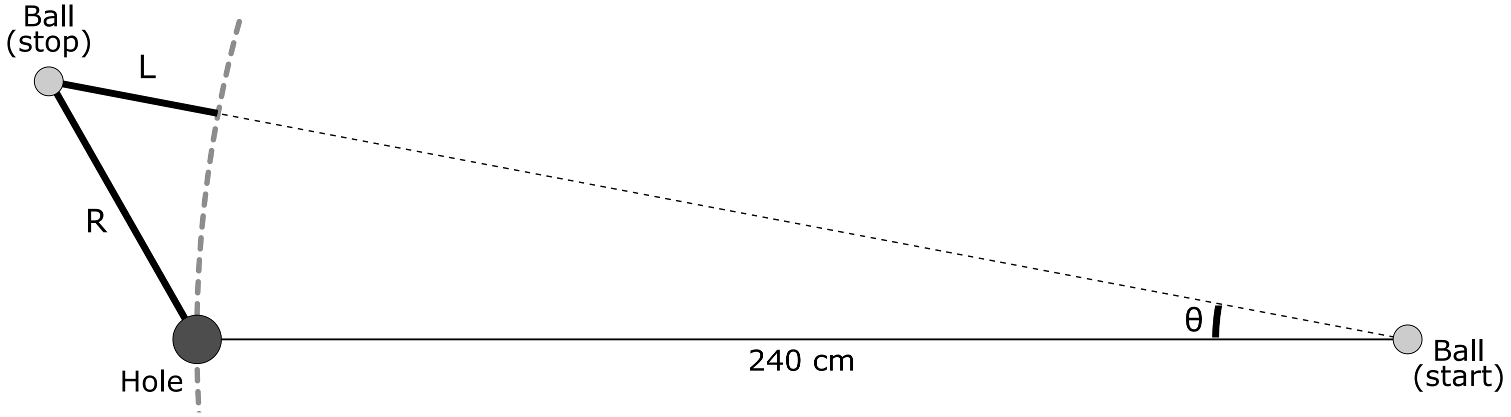


*Figure S1.*
Performance errors for a missed putt. Radial error ("R", in cm): hole to ball (stop) distance. Angle error ("θ", in degrees): absolute value of the angle between the ball (start) to hole and the ball (start) to ball (stop) lines. Length error ("L", in cm): absolute value of the ball (start) to hole distance minus the ball (start) to ball (stop) distance. All errors were 0 when the ball was holed.
