# Supplementary material for: Practice Makes Efficient: Cortical Alpha Oscillations Are Associated With Improved Golf Putting Performance
Source: Sport Exerc Perform Psychol. 2016 Nov 28;6(1):89–102. doi: 10.1037/spy0000077 (PMC5506342; doi:10.1037/spy0000077)
Supplement: Supplementary file 5 [file FigureS6.pdf]

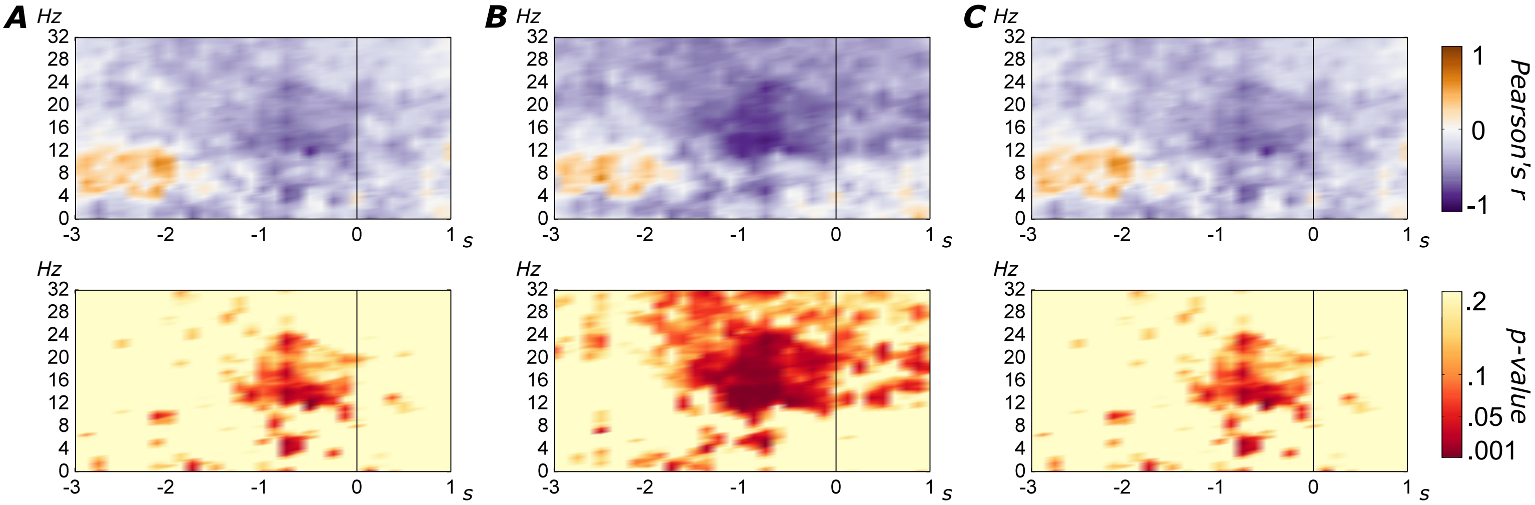

Figure S6.

Time-frequency plots representing Pearson's correlations conducted on the inter-session change scores between the left temporal alpha power (10·log10(μV²)) and the radial **(A)**, angle **(B)**, and length **(C)** error, as a function of time (–3 to +1 s) and frequency (0 to 32 Hz).
