# Supplementary material for: Practice Makes Efficient: Cortical Alpha Oscillations Are Associated With Improved Golf Putting Performance
Source: Sport Exerc Perform Psychol. 2016 Nov 28;6(1):89–102. doi: 10.1037/spy0000077 (PMC5506342; doi:10.1037/spy0000077)
Supplement: Supplementary file 6 [file FigureS7.pdf]

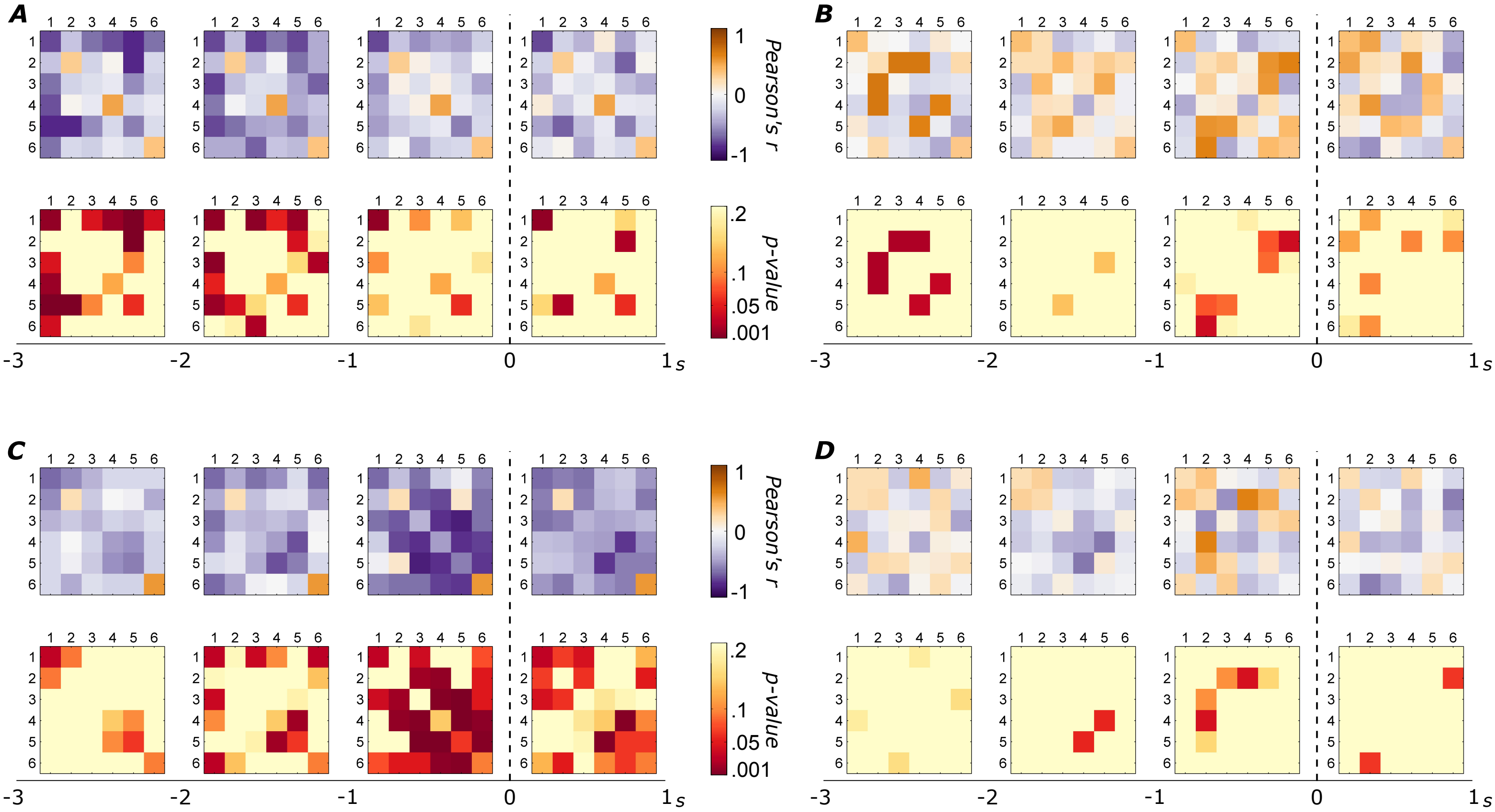

Figure S7.
Matrix maps representing Pearson's correlations conducted on the inter-session change scores between the number of holed putts and alpha ISPC **(A)** and imISPC **(B)**, and between conscious processing and alpha ISPC **(C)** and imISPC **(D)**, as a function of time (−3 to +1 s), and ROI pairs (1 = left temporal, 2 = left central, 3 = frontal, 4 = right central, 5 = right temporal, 6 = occipital).
