# Supplementary material for: Practice Makes Efficient: Cortical Alpha Oscillations Are Associated With Improved Golf Putting Performance
Source: Sport Exerc Perform Psychol. 2016 Nov 28;6(1):89–102. doi: 10.1037/spy0000077 (PMC5506342; doi:10.1037/spy0000077)
Supplement: Supplementary file 7 [file FigureS4.pdf]

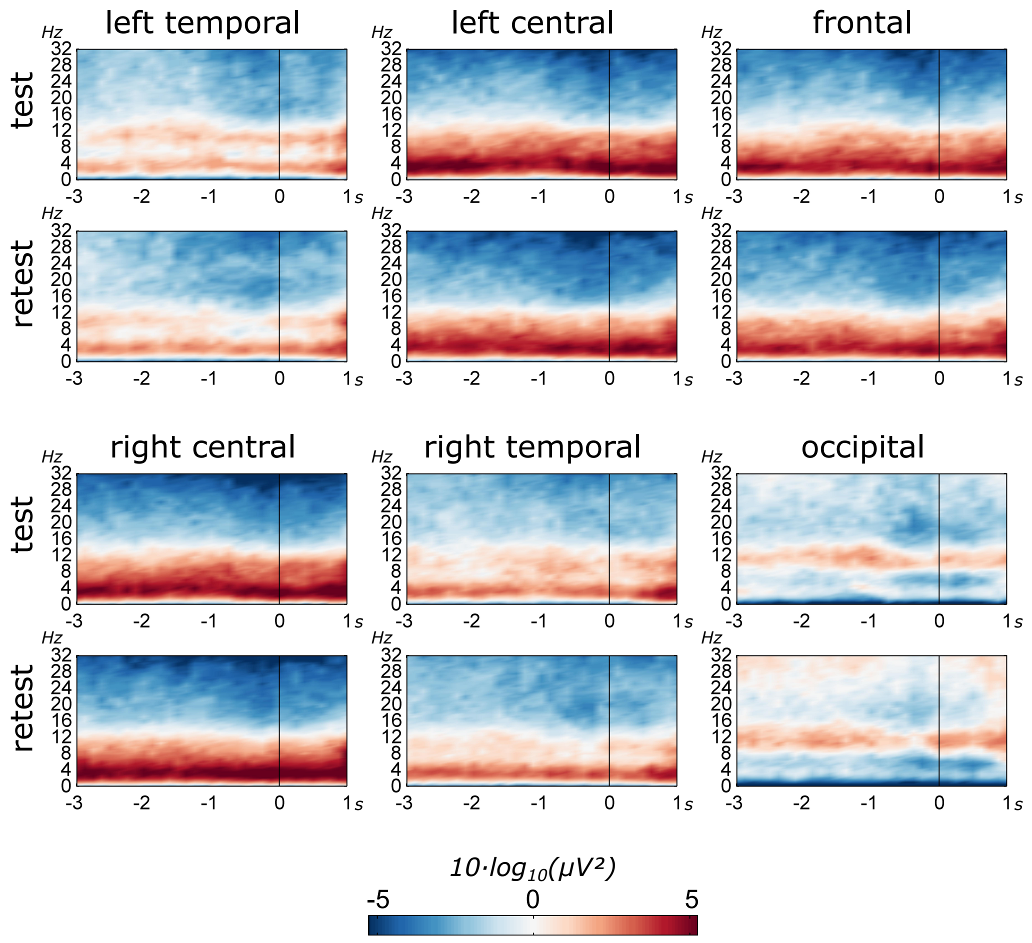

Figure S4.
Time-frequency plots representing power (10·log10(μV²)) averaged across participants, as a function of session (test, retest), time (–3 to +1 s), and frequency (0 to 32 Hz), in the six regions. Values within each participant and region were scaled by their median, prior to log-transformation, in order to represent all frequencies on the same scale for each region.
