# Supplementary material for: Practice Makes Efficient: Cortical Alpha Oscillations Are Associated With Improved Golf Putting Performance
Source: Sport Exerc Perform Psychol. 2016 Nov 28;6(1):89–102. doi: 10.1037/spy0000077 (PMC5506342; doi:10.1037/spy0000077)
Supplement: Supplementary file 9 [file FigureS8.pdf]

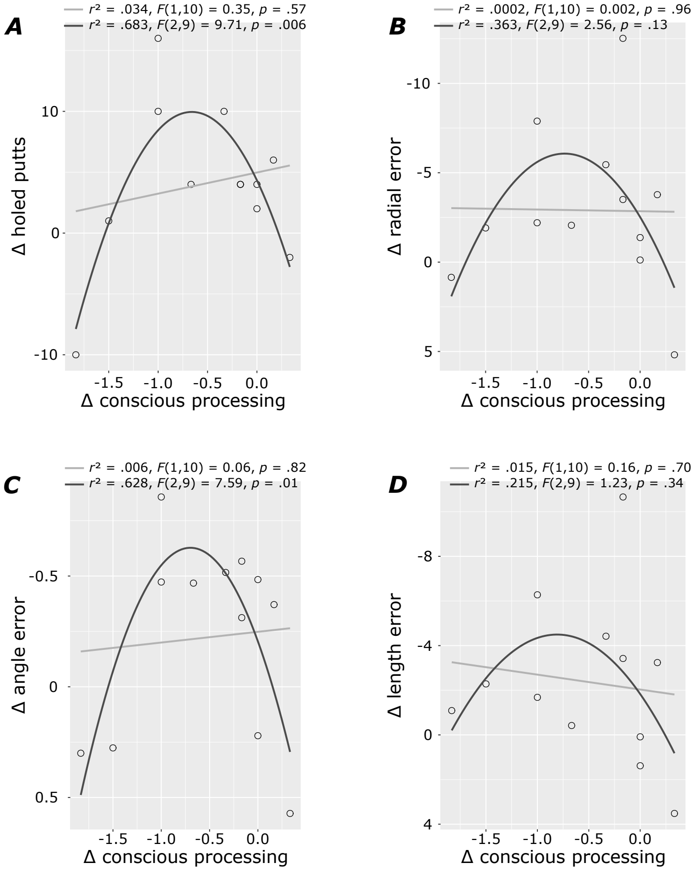

Figure S8.

Scatter plots representing Pearson's correlations conducted on the inter-session change scores between conscious processing and the number of holed putts **(A)**, radial **(B)**, angle **(C)**, and length **(D)** error. The grey and black lines represent the linear ($y = a + bx$) and the quadratic ($y = a + bx + cx^2$) best fit of the data, respectively. Statistics are reported for the goodness of fit for linear and quadratic models. The y-axis for the radial, angle, and length errors was inverted to facilitate comparisons with the number of holed putts.
